# Interstitial fluid: the overlooked component of the tumor microenvironment?

**DOI:** 10.1186/1755-1536-3-12

**Published:** 2010-07-23

**Authors:** Helge Wiig, Olav Tenstad, Per Ole Iversen, Raghu Kalluri, Rolf Bjerkvig

**Affiliations:** 1 Department of Biomedicine, University of Bergen, Bergen, Norway; 2 Division of Matrix Biology, Department of Medicine, Beth Israel Deaconess Medical Center and Harvard Medical School, MA, USA; 3 Department of Nutrition, Institute of Basic Medical Sciences, Faculty of Medicine, University of Oslo, Oslo, Norway

## Abstract

**Background:**

The interstitium, situated between the blood and lymph vessels and the cells, consists of a solid or matrix phase and a fluid phase, together constituting the tissue microenvironment. Here we focus on the interstitial fluid phase of tumors, *i.e*., the fluid bathing the tumor and stromal cells. Novel knowledge on this compartment may provide important insight into how tumors develop and how they respond to therapy.

**Results:**

We discuss available techniques for interstitial fluid isolation and implications of recent findings with respect to transcapillary fluid balance and uptake of macromolecular therapeutic agents. By the development of new methods it is emerging that local gradients exist in signaling substances from neoplastic tissue to plasma. Such gradients may provide new insight into the biology of tumors and mechanistic aspects linked to therapy. The emergence of sensitive proteomic technologies has made the interstitial fluid compartment in general and that of tumors in particular a highly valuable source for tissue-specific proteins that may serve as biomarker candidates. Potential biomarkers will appear locally at high concentrations in the tissue of interest and will eventually appear in the plasma, where they are diluted.

**Conclusions:**

Access to fluid that reliably reflects the local microenvironment enables us to identify substances that can be used in early detection and monitoring of disease.

## Introduction

The interstitial space consists of connective and supporting tissues of the body and is located outside the blood and lymphatic vessels and parenchymal cells. Essentially the interstitium can be divided into two compartments: the interstitial fluid and the structural molecules of the interstitial or the extracellular matrix (ECM). In the present review we focus on the interstitial fluid phase, which contains an array of regulatory molecules defining the physical and biochemical microenvironment of cells. All organs have an interstitium, although at a variable amount. The structure and composition of the interstitium differs considerably depending on the mother organ. Interstitial water with its solutes, the interstitial fluid volume, serves as a transport medium for nutrients and waste products between cells and capillary blood and also harbors various signaling substances that are either produced locally or brought to the organ by the circulation.

Recently there has been a renewed interest in the tumor microenvironment because of its role in tumor growth and metastasis. The tumor microenvironment can be defined as the insoluble elements of the ECM, the stroma with its cellular elements such as fibroblasts and immune cells and the fluid phase of dissolved substances. Traditionally the focus has been on the stroma and the cellular elements of the tumor, whereas here we focus on the fluid phase that may be thought of as a "misconsidered component of the internal milieu of a solid tumor" [1].

We discuss recent data on the isolation of interstitial fluid and what can be learned from analyses of such fluid regarding local production of signaling substances and potential biomarkers as well as the level of interstitial fluid colloid osmotic pressure, one of the determinants of transcapillary fluid exchange. For a broader description of the latter topic, the reader is referred to reviews on transcapillary fluid exchange and interstitial fluid volume regulation [2-9]. The composition and structure of the interstitium has been the topic of several extensive reviews [10-15], and accordingly this topic is not discussed here.

## The tumor interstitium

**Figure 1:**
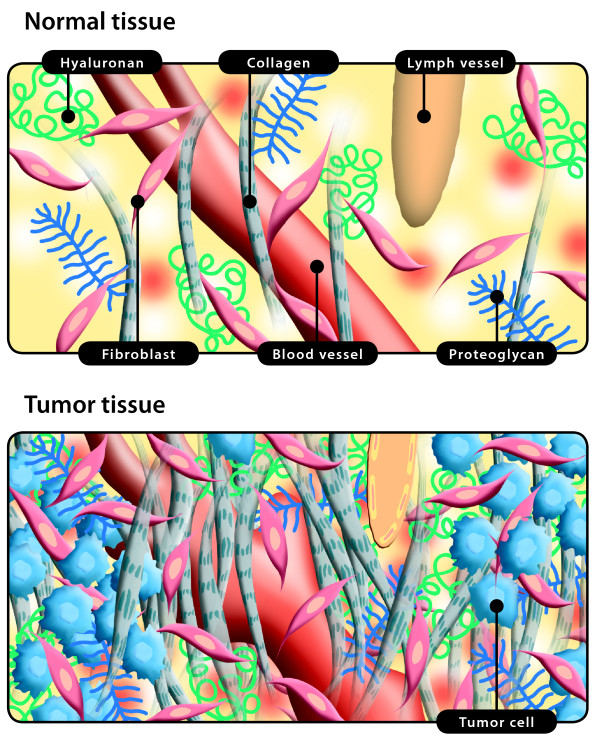
**The interstitial space in normal tissue and tumors**. *Top*: The interstitium (*i.e*., loose connective tissue outside the blood and lymph vessels) in normal tissue consists of interstitial fluid and a solid extracellular matrix (ECM) again consisting of collagen fibers, glycosaminoglycans, *i.e*., hyaluronan and proteoglycans and fibroblasts. Notice the lymphatic vessel that is filled and drains filtered fluid and immune cells. *Bottom*: The interstitium in tumors is more disorganized than in normal tissue, and tumors have a so-called reactive stroma. A normal stroma in most organs contains a minimal number of fibroblasts, whereas reactive stroma is associated with an increased number of fibroblasts, enhanced capillary density and irregular blood vessels that have high microvascular permeability, again resulting in extravasation of plasma proteins such as fibrin, which in turn attract an influx of fibroblasts, inflammatory cells and endothelial cells. Compared with non-neoplastic tissue, the tumor stroma contains increased amounts of collagen having variable fiber size, proteoglycans and glycosaminoglycans, especially hyaluronan and chrondroitin sulfate. Tumors have lymphatics, at least in the periphery, but lymphatics may be compressed (pictured as a flattened lymph vessel) and thus nonfunctional.

Although the tumor interstitium consists of the same components as the interstitia of normal tissues, *i.e*., an extracellular or interstitial matrix composed of solid elements constituting the framework of the tumor and a fluid phase constituting the interstitial fluid; it has its special features that are discussed briefly here. Before we turn to the interstitial/extracellular fluid phase, we summarize some common features of the ECM or solid phase in tumors compared to normal interstitium, as schematized in Figure 1. We do not discuss the properties of the tumor stroma, which has been the topic of several extensive recent reviews [16-22]. As stated by Kalluri and Zeisberg [18], there is considerable interest in understanding the differences between a normal stroma and a reactive tumor stroma. The normal stroma in most organs contains a minimal number of fibroblasts, whereas a reactive stroma is associated with an increased number of fibroblasts, enhanced capillary density, and type I collagen and fibrin deposition. A key factor in the formation of the reactive stroma is the vascular endothelial growth factor (VEGF) [23], either released by the cancer cells themselves or by fibroblasts, or inflammatory cells [24]. Increased levels of VEGF results in a high microvascular permeability that causes extravasation of plasma proteins such as fibrin, which in turn attract an influx of fibroblasts, inflammatory cells and endothelial cells [25,26]. The resulting reaction has similarities to wound healing where the cells produce an ECM rich in fibronectin and type I collagen and thus influence the composition of the stroma but also contribute to the process of initiating tumor angiogenesis [26,27]. Compared with non-neoplastic tissue, the tumor stroma contains increased amounts of collagens, proteoglycans and glycosaminoglycans, especially hyaluronan and chrondroitin sulfate, [*e.g*., [28-30]], as reviewed in [31]. These cellular responses are parallel to wound healing; the difference is that the generation of tumor stroma may be considered dysregulated wound healing [27].

## Tumor interstitial fluid

The abnormal interstitium representing the microenvironment of the tumor cells has puzzled researchers for many years and continues to do so. Recently, an increased interest in the tumor microenvironment and its influence on cancer progression has turned attention toward this compartment (*e.g*., [18,19,22,32-34]). One may therefore envisage that access to tumor interstitial fluid (TIF), *i.e*., the fluid bathing the tumor and stroma cells, is of considerable importance to understand how tumors develop and progress. The properties of the tumor interstitium, *e.g*., the high vascularity and cellularity, represent major challenges when trying to gain access to the TIF.

### Methods of fluid isolation

To quantify one of the determinants of the transcapillary fluid balance, the interstitial fluid protein concentration, and thus interstitial fluid colloid osmotic pressure as well as the tissue fluid concentration of signaling substances and molecules that may reflect local cellular processes, it is imperative to have the appropriate methodologies that provide reliable and representative native interstitial fluid.

In tumors, there is no uniform agreement as to which method to use to isolate TIF. As discussed above, lymph has been generally accepted as a measure of interstitial fluid [3]. Although many studies have shown that lymph vessels are present in tumor tissue (for review, see [35-38]), these vessels appear to be nonfunctional, *i.e*., not draining any fluid, at least in central tumor areas [37,39]. In addition, tumor lymphatics may not be cannulated, making lymph sampling inapplicable in this tissue, showing the need for alternative methods in tumors. We therefore discuss the available methods and their strengths and weaknesses from a tumor point of view (summarized in Table 1) before we turn to a discussion on recent data on composition of the interstitial fluid.

**Table 1:** Methods for tumor interstitial fluid isolation

Method	How performed	What was sampled	Advantages	Disadvantages	Remarks	References
Glass capillaries	Insertion by blunt dissection *in vivo*	Fluid from tumor periphery or sectioned surface	*In vivo *native fluid	Bleeding and inflammation, cellular disruption	High level of intracellular enzymes in isolated fluid	[40]

Implantable chambers	Chronically implanted	Fluid draining from central part of tumor	*In vivo *native fluid, continuous and repeated sampling	Inflammation in early phases, scar formation	Requires chronic restraining of animal	[41]

Implanted wicks	Implanted acutely or chronically	Fluid absorbed into wicks during implantation	*In vivo*, native fluid	Bleeding and inflammation, cellular disruption	Chronic implantation more representative than acute	[43]

Microdialysis	Insertion of semipermeable membrane	Substances diffusing across membrane	*In vivo *continuous and repeated sampling	Inflammation, incomplete recovery, dilute fluid	Recovery especially low for macromolecules	Reviewed in [45]

Capillary ultrafiltration	Negative pressure applied to semipermeable membrane	Substances transported by bulk flow across membrane	*In vivo *continuous and repeated sampling	Inflammation, incomplete recovery	Recovery especially low for macromolecules	[60]

Tissue centrifugation	Exposure of excised tissue to increased G-force	Fluid from tumor periphery or bone marrow	Native fluid	*Ex vivo *single samples	Composition validated by extracellular tracers	[61,77]

Tissue elution	Elution of minced tissue	Substances dissolved in elution buffer	Technically easy	*Ex vivo *single samples, dilute fluid	Contamination by intracellular proteins likely	[67]

#### Glass capillaries

The glass capillary method for TIF isolation was described by Sylven and Bois [40]. They noted that the tumor periphery was rich in edema-like interstitial fluid, and isolated such fluid by inserting capillaries, 0.1-0.6 mm in diameter, into pouches made by blunt dissection in and along the periphery of the tumor or into sectioned tumor surface. The sampling procedure did not allow exact localization of the site of origin of the fluid, and Sylven and Bois acknowledged that it was possible that normal tissue fluid could also be included. Another obvious problem is an inherent tendency for cellular and vascular leakage that is likely to occur during sampling. The isolated fluid contained very high levels of intracellular enzymes, which indicates that the fluid isolated this way is a mixture of interstitial and intracellular fluid.

#### Implantable chambers

Much of our knowledge on TIF derives from experiments performed by Gullino *et al. *[41] in rats bearing various tumors using implantable chambers. The chambers were either inserted into a growing tumor or enclosed in it by allowing a tumor to grow around the chamber. The chamber was separated from tumor tissue by a porous membrane. Fluid draining into the chamber could be collected by a catheter draining the cavity. After a period, necrosis developed and some hemorrhages appeared, but there was a definite interval when the chamber was enclosed by neoplastic cells and a low amount of necrosis that was used for fluid sampling. A main advantage with this approach is that fluid can be harvested for longer periods, *e.g*., to follow effects of therapy. Many of the potential problems with the method have been discussed by Jain [42]. One obvious issue is the inflammatory reaction and subsequent scarring that is induced by chamber insertion.

#### Implanted wicks

As for skin and muscle (for review, see [3]), wicks have been implanted in various types of solid tumors in mice to isolate interstitial fluid. Stohrer *et al. *[43] implanted saline-soaked wicks directly into established tumors (termed *acute wicks*) or simultaneously with tumor implantation (termed *chronic wicks*) and characterized the isolated fluid. In addition to evaluating the effect of acute and chronic insertion, they evaluated the effect of both implantation time and tumor type. They concluded that acute wick implantation requires a long time for equilibration (*i.e*., >120 min) and that chronic wicks should be preferred to avoid bleeding and cellular damage. Furthermore, as pointed out by Stohrer *et al*., cellular proteins deriving from necrotic cells or cells disrupted during wick removal may enter the wick fluid and cause a higher protein concentration (and higher colloid osmotic pressure). As a result, the protein distribution pattern will deviate from that of undisturbed tumor interstitial fluid, *i.e*., mainly proteins with lower molecular weight (MW) than albumin, *e.g*., as observed in skeletal muscle [44].

#### Microdialysis

The microdialysis technique, based on diffusion of analytes across a semipermeable membrane, has been applied extensively to study TIF (for reviews, see [45-52]). With this technique it is possible to sample endogenous and exogenous substances from the extracellular space, mainly small molecular species. At present, there has been an increased interest in using the technique in pharmacokinetic and pharmacodynamic studies [48,49,53], but it has also been applied to study peptides and proteins dissolved in the interstitial fluid (for recent reviews see, *e.g*., [54,55]). In such a context the major problems with the method is incomplete recovery of substances and also the potential inflammatory reactions resulting from insertion of the probe, and as pointed out by Clough [55], it is therefore unlikely that the dialysate reflects a representative concentration of the interstitial fluid.

#### Capillary ultrafiltration

Ultrafiltration has traditionally been used for separation or purification of chemicals, but this technique has also been applied to sample tissue fluid by implanting capillary ultrafiltration probes in vivo (reviewed in [56]). With this method fluid is sampled using negative pressure as a driving force, and the restriction induced by the semipermeable membrane affects the size of molecules that are allowed in the ultrafiltrate sample. As for microdialysis, the recovery for small molecules is close to 100%, whereas in vitro recovery for albumin has been found to be 74% [57], suggesting that ultrafiltration can be used for interstitial fluid sampling. The technique has also been used for collection of tissue fluid from skin [58,59] and fibrosarcomas in mice [60] using membranes with MW cutoff of 400 kDa to allow for sampling of proteins secreted to the interstitial fluid ("secretome"). The protein concentration in the sampled fluid is, however, very low compared to that obtained with other methods (1/300 or lower for skin or <1/1000 for tumors; see section "Composition of tumor interstitial fluid" and Table 2). These results suggest that proteins are sieved off at the capillary membrane, in the tissue or at the tissue-membrane interface during ultrafiltration, and accordingly that ultrafiltration fluid does not represent interstitial fluid composition.

#### Tissue centrifugation

**Figure 2:**
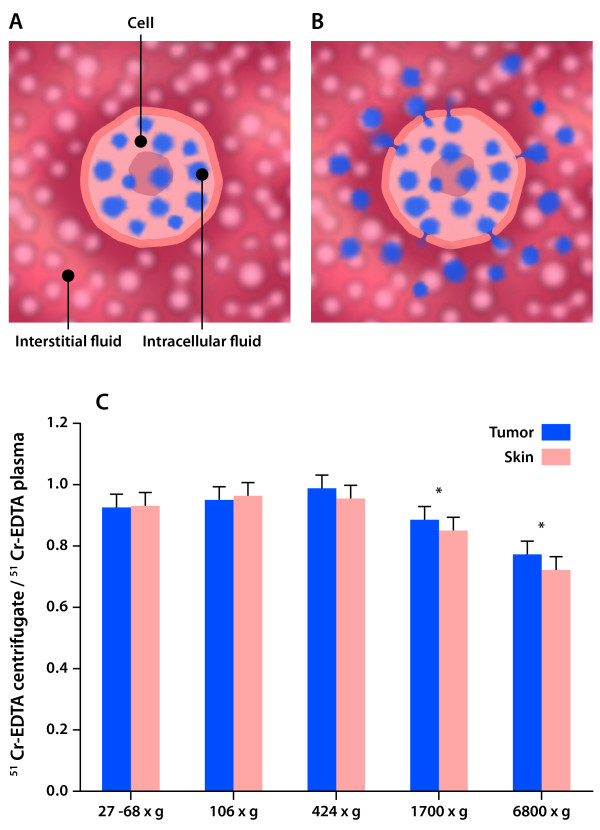
**Determination of possible contamination of interstitial fluid**. (A) A tracer that does not pass intracellularly is equilibrated in the extracellular fluid phase. (B) If undiluted interstitial fluid (IF) is isolated, the concentration in IF and plasma (P) will be equal, *i.e*., IF/P = 1.0. If, however, intracellular fluid (with dissolved proteins) not containing tracer is added to the interstitial fluid during the isolation process (*e.g*., centrifugation), the IF will become diluted and as a consequence IF/P <1.0. (C) Tissue-to-plasma distribution ratios (IF/P) of the extracellular tracer ^51^Cr-ethylenediaminetetraacetic acid (EDTA) as a function of G-force for interstitial fluid isolated by centrifugation from intact tumor and skin (means ± SE). For *g *< 424, the IF/P ratio was not significantly different from 1.0, suggesting negligible dilution and thus contamination of IF. An IF/P <1.0 for *g *> 424 (*P < 0.05 for tumor as well as skin) suggested contamination of IF with intracellular water and proteins (Data in Figure 2C modified from [61]).

Recently, we described a centrifugation method for tumor interstitial fluid isolation [61] based on experiments having shown that tissue fluid could be isolated from cornea [62] and tail tendon [63,64] by exposing tissue to increased G-force. Crucial questions are whether the isolated fluid represents undisturbed interstitial fluid and whether there is contamination with cell fluid. Cell compression during centrifugation may lead to extrusion of cellular fluid, resulting in the isolation of a mixture of interstitial and cytoplasmic fluid. This question has been addressed in a study in rats bearing chemically induced mammary carcinomas using the extracellular tracer ^51^Cr-ethylenediaminetetraacetic acid (EDTA) as a probe to show possible 'contamination' of cellular fluid. Addition of tumor cell fluid to the centrifuged volume should thus show up as a reduced ^51^Cr-EDTA concentration in the centrifugate relative to plasma (Figure 2). The finding of a ratio in peripheral tumor not significantly different from 1.0 for *g* ≤ 424 suggested no dilution of extracellular fluid, indicating that the isolated fluid is representative for TIF provided a G-force in this range. The procedure was later found suitable for interstitial fluid isolation in other tumor models [65,66].

#### Tissue elution

Recently, in a search for novel biomarkers, Celis *et al. *[67] proposed that tissue elution would be a suitable method to isolate tumor interstitial fluid. They used clean, fresh biopsies obtained from women with invasive breast cancer. Biopsies were cut into small pieces (1-3 mm^3^) that were washed carefully and placed in tubes containing phosphate-buffered saline. After incubation or elution for 1 h followed by centrifugation, the supernatant was collected and named tumor interstitial fluid. Later, they used the same elution method to isolate fat interstitial fluid [68]. In the tumor study, they found that the TIF contained some major serum proteins as might be expected, but that its overall protein profile was remarkably different from that of serum. Clearly, sectioning of cell-rich tumors into small pieces results in sectioning of an unknown fraction of cells and addition of cell fluid to the eluate.

## Composition of tumor interstitial fluid

The functional importance of the TIF has been acknowledged in earlier studies by Jain [42], and it is therefore surprising that there has been little focus on the TIF compartment. We here briefly discuss old data and focus on new developments. In doing so, we discuss the data in light of the limitations inherent for the methods used for fluid isolation.

Gullino *et al. *[41,69] were the first to measure the concentration of various low molecular weight solutes, and in Table 2 we have summarized data on some characteristics of TIF. When compared to plasma and subcutaneous interstitial fluid, TIF has high H^+^, CO_2_, and lactic acid and low glucose and O_2_, probably a result of tumor metabolism [42]. Of note, the pH of the TIF was 0.2-0.4 units lower and fell even more with increasing tumor size [70], the P_CO2 _was 16-39 mmHg higher, and the concentration of bicarbonate was 4-6 mmol/l higher when compared to afferent plasma.

**Table 2:** Composition of interstitial fluid in tumors

Tumor type	Host	Protein, mg/ml			COP, mm Hg			pH		Lactic acid, mg/l		Reference
		**TIF**	**Subcutis**	**Plasma**	**TIF**	**Subcutis**	**Plasma**	**TIF**	**Plasma (arterial)**	**TIF**	**Plasma**	

Carcinoma (Walker 256)	Rat	32 ± 1	41 ± 2	48 ± 1				7.044 ±0.044	7.313 ± 0.041	16.1 ± 1.1	9.5 ± 0.9	[41,95]

Mammary carcinoma	Mouse	54		55 - 60								[40]

Carcinoma (Walker 256)	Rat							6.98 ± 0.13		15	15	[70]

Colon adenocarcinoma (LS174T)	Mouse				16.7 ± 3.0							[43]
Small cell lung carcinoma (54A)					21.1 ± 2.8	8.2 ±2.3	20.0 ± 1.6					

Mammary carcinoma (chemically induced)	Rat	44.7		54.9	16.6 ± 1.0	13.8 ±1.0	20.5 ±0.8					[61]

COP: Colloid osmotic pressure, TIF: Tumor interstitial fluid. Empty cells in table: Value not determined

There has, however, been some development during recent years regarding the macromolecular composition of TIF that is of importance for our understanding of transcapillary fluid balance in tumors. The transcapillary fluid exchange is determined in tumors as in normal tissue by the Staling principle, *i.e*., by the net filtration pressure being the difference between the hydrostatic and colloid osmotic pressure acting across the capillary wall.

Whereas there are numerous studies where tumor interstitial fluid pressure has been measured and found to be elevated (for review, see [71]); in two studies only has colloid osmotic pressure (COP) been measured in TIF (Table 2). Stohrer *et al. *[43] determined COP_TIF _in four different human tumor xenografts in mice using implanted wicks. Using chronic wicks (see section "Implanted wicks" above), they found a generally higher pressure in TIF than in subcutaneous tissue. In three of the four tumor types, COP in TIF was not significantly different from plasma (or actually tended to be higher), whereas in a colon adenocarcinoma (LS174T), the COP was 82% of that in plasma and the COP in subcutaneous interstitial fluid was 41% of the plasma value. In a recent study, we measured COP in TIF isolated by centrifugation of excised chemically induced mammary carcinomas [61], and we found that the TIF/plasma COP ratio was 0.75-0.79, again significantly higher than the corresponding subcutaneous ratio of 0.60 and close to the value for LS174T colon adenocarcinomas. The high COP observed in these two studies corresponds well to the high protein concentration relative to plasma of 0.8-1.0 found by Sylven *et al. *[40], although the data from Gullino *et al. *[41] suggest lower COP in TIF in their model. Thus, although some caution must be expressed regarding potential contamination of intracellular and plasma proteins discussed for capillary and wick sampling above (see sections "Glass capillaries" and "Implanted wicks"), all data suggest that there is a high colloid osmotic pressure and protein concentration in TIF.

The observed protein distribution pattern in interstitial fluid may also be of interest since this pattern may have implications for the interpretation of potential biomarker candidates that are discussed in the following section. Gullino *et al. *[41] subjected TIF to paper electrophoresis and found an albumin-to-globulin ratio of TIF similar to that of afferent blood. A somewhat different picture was observed by Stohrer *et al. *[43] after separation by sodium dodecyl sulfate-gel electrophoresis. For proteins 25-75 kDa, the concentration in TIF and plasma was not significantly different, whereas for proteins with MW <25 kDa the concentration in TIF was on average 2.4 times higher than in plasma. As noted by Stohrer *et al. *[43], these smaller proteins can be breakdown products from necrotic areas and tumor-derived cell proteins as well as cellular enzymes [40]. We compared the gel chromatography elution patterns for TIF with that of plasma [61], and whereas the patterns for albumin (MW ~69 kDa) and larger molecules were similar, there was a larger fraction of molecules eluting in the lower MW region, in agreement with data from Stohrer *et al. *[43] discussed above. Clearly there is a need for identification of proteins in TIF to better characterize the microenvironment and thus to understand local signaling events as well as to search for potential biomarkers.

## Biological implications of recent studies on interstitial fluid

Adding to the value of identifying "new" substances in the interstitium, *e.g*., using proteomic approaches, it is also important to quantify known bioactive compounds in the interstitial fluid. Numerous growth factors and cytokines are associated with and sequestered in the ECM (*e.g*., [72-75]). Posttranslational modifications may occur in this compartment, and to be able to understand biological processes, it may be of importance to monitor biomolecules in the compartment where they are biologically active, *i.e*., in the interstitial fluid rather than at a gene level as elegantly shown by Garvin and Dabrosin [76]. In a mammary cancer model they demonstrated that although estradiol and the antiandrogen tamoxifen increased mRNA and intracellular VEGF protein, the secreted VEGF to the extracellular phase, and thereby angiogenesis, was decreased by the latter substance. Such observations highlight the importance of studying signaling substances in the interstitial fluid phase of the target organ and also show the importance of quantification of substances in the microenvironment. 

**Figure 3:**
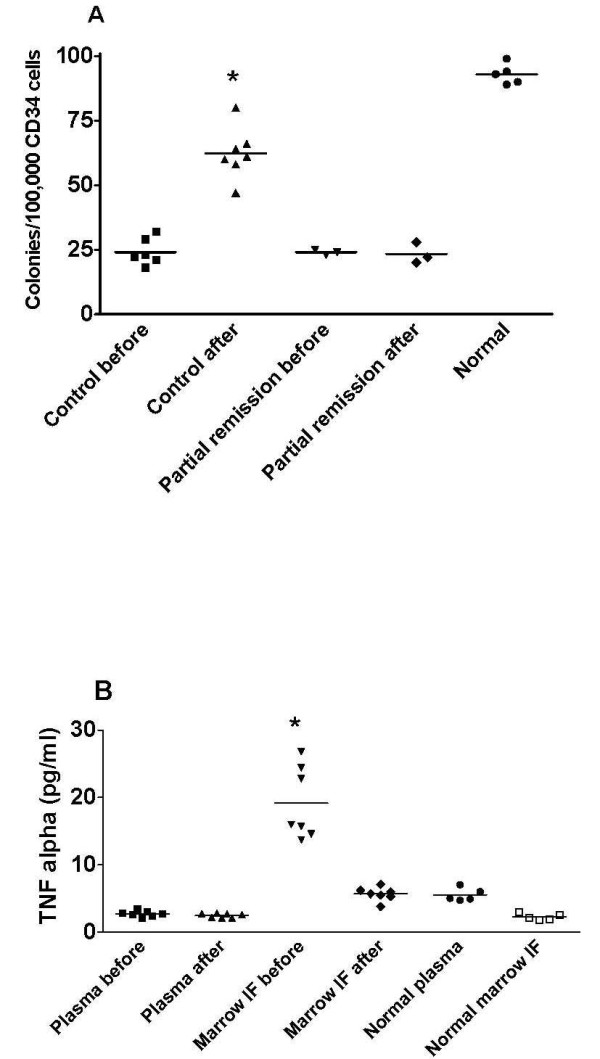
**Signaling substances in bone marrow interstitial fluid (BMIF) in leukemia**. (A) Growth of normal bone marrow progenitor cells (CD34 cells) added BMIF from patients with acute myeloid leukemia (AML) before and after induction chemotherapy. A marked increase in colony numbers (*P < 0.05) was noted upon this initial treatment in 7 of 10 patients with AML entering complete remission. BMIF from the remaining three AML patients that only entered partial remission yielded similar colony numbers before and after induction chemotherapy. Colony formation in the presence of normal BMIF is shown for comparison. (B) Tumor necrosis factor (TNF)-α concentrations in plasma and BMIF before and after induction chemotherapy in 7 of 10 patients with AML entering complete remission. This treatment resulted in a substantial fall in TNF-α concentration (*P < 0.05) in the BMIF, but not in plasma. Normal plasma and BMIF values are shown for comparison. Individual values are shown. Horizontal lines refer to means. Modified from [78].

Having demonstrated that interstitial fluid could be isolated from bone marrow in rats and humans by centrifugation [77], we then isolated bone marrow interstitial fluid (BMIF) from patients with acute myeloid leukemia (AML) at the time of diagnosis and 2-4 weeks after the start of induction therapy [78]. We found that AML-derived BMIF, but not plasma, repressed hematopoietic cell growth and that this effect was lost by successful induction treatment (Figure 3). Tumor necrosis factor α and adiponectin concentrations were higher in BMIF showing local production, and our experiments [78] suggested that these two cytokines had a mechanistic role in the disease process. Whereas plasma levels of these cytokines were unaffected by therapy, the levels fell significantly in BMIF in patients entering remission, showing that quantification of substances in interstitial fluid may give important information on disease progression. Two recent studies [79,80] have shown the therapeutic importance of targeting syndecan-1 heparan sulfate proteoglycan in the local microenvironment and not in plasma in hematological cancers.

### The microenvironment as a potential target for identification of biomarkers

The advances of mass spectrometry techniques combined with bioinformatics allow detection, identification and quantification of numerous peptides and proteins in biological samples (*e.g*. [81-86]). The emergence of these technologies and the possibility of isolating fluid deriving from a specific tissue environment offer new avenues for detection of tissue-specific biomarkers, notably for tumors. Biomarkers may be defined as molecular indicators whose presence or metabolism correlates with important disease-related processes and/or disease outcomes [87] and may be important for detection of risk, early disease and response to treatment. An attractive source for biomarkers is fluids that can be sampled noninvasively, and in addition to plasma/serum, such studies have been performed on urine, cerebrospinal fluid, bronchoalveolar lavage fluid, synovial fluid, nipple aspirate fluid, tear fluid and amnion fluid (reviewed in [88]). One general problem is the complexity of the fluids sampled and the dynamic range in the proteins sampled. This applies especially to plasma/serum, and therefore techniques have emerged to enrich and study subproteomes to reduce the complexity and to be able to detect low abundant proteins [89,90]. Still, even when studying subproteomes of plasma/serum, the potential biomarkers deriving from a local disease process such as solid cancer will be diluted in a substantial volume, which represents the sum of the cellular processes of the body. The chances of detecting disease-specific biomarkers will therefore be greater if the search is performed closer to the disease process, *i.e*., in the tissue microenvironment. Tumor cells are known to release specific substances that disrupt the tissue and elicit local responses, and it is likely that candidates can be found locally in the tumor cell secretome, *i.e*., specific substances secreted by tumor cells [91-94]. For this purpose it is necessary to have a technique that reflects the true interstitial fluid.

## Summary and perspectives

Although substantial interest has been devoted to the ECM, the same cannot be said about the interstitial or extracellular fluid phase. Here we have focused on the interstitial fluid phase of tumors, and although the importance of this type of study in normal as well as neoplastic tissues was emphasized more than 20 years ago [42], there have been surprisingly few studies on this topic in tumors since the classical studies by Gullino *et al. *[41] more than 40 years ago. More recently it has been established that the colloid osmotic pressure gradient across the tumor microvascular wall is low, consistent with the findings of increased permeability of tumor vessels. With the development of new methods, local cytokine gradients between tumor and plasma have been demonstrated that may result in new insight in tumor biology. The emergence of proteomic technologies makes interstitial fluid in general, but particularly that from tumors, a very valuable source for biomarkers. As we have discussed, a major challenge in this quest is to ascertain that the source fluid derives from the interstitial fluid, but if we can, the potential of such an approach is substantial.

## Competing interests

The authors declare that they have no competing interests.

## Authors' contributions

HW conceived of the study and wrote the paper, OT, POI, RK and RB participated in its design and helped to draft the manuscript. All authors read and approved the final manuscript.
